# The Departmental Report on Tuberculosis

**Published:** 1913-03-22

**Authors:** 


### THE DEPARTMENTAL REPORT ON TUBERCULOSIS.

The Final Report of the Departmental Committee on Tuberculosis appointed more than twelve months ago deals largely with the measures that the Committee thinks should be taken for the prevention of tuberculosis in general. Incidentally the Committee suggests a scheme for spending the money which is available, under the terms of the National Insurance Act, for research purposes. From an expert scientific point of view the report contains nothing that is original or strikingly interesting. The premise that bovine tuberculosis is of essential importance in the causation of the disease in man is fully accepted by the Committee, and the suggestion is thrown out that one of the best lines of attack would be to eradicate bovine tuberculosis completely. No one can have the slightest objection to that suggestion, which, we may point out, has already been adopted in Denmark, where the Bang system is said to work well.

But nevertheless we fear that the Committee has not fully realised that there are plenty of other factors which are possibly of equally vital importance and which should be dealt with. The slaughter of infected cows and the destruction of tainted meat are steps in the right direction, but they must of necessity be steps co-ordinated to a vigorous hygienic campaign in which the main planks are sanitary housing of the poorer classes, the prevention of overcrowding, and the strict supervision and regulation of the many dangerous trades and occupations which still flourish in every industrial community in these islands. We cordially welcome the Committee's recommendation that organised research should be carried out in the investigation of what may be called the problems of tuberculosis. More than once we have pointed out the urgent necessity of investigating certain points in connection with the disease, particularly with regard to the incidence of consumption in children. Such investigation can be carried out only by men and women who have been properly trained and who work in close co-partnership with lay workers who are fully acquainted with the environmental conditions of the community. Above all, it is necessary that the statistics of these expert workers shall be subject to a close scrutiny by impartial statistical experts. We have seen the importance of such impartial investigation and scrutiny in the valuable criticisms supplied by Professor Pearson and his co-workers, and it is safe to say that the expenditure of money to secure such a staff will be a good investment.

The establishment of a central bureau is another important suggestion which is well worth adoption. Such a central organisation can do excellent work for the public as well as for the individual investigator, so long as it is adequately financed and not handicapped by a chronic want of funds. The annual award of a large prize for the best research work hardly appeals to us. What is wanted is not to give a monetary recompense to individual workers who have accomplished something—since for them there are always the professional prizes which mean more than mere money can counterpoise—but financial aid to those who wish to carry on research work and who have not the private means to do so. In our opinion it would be better to assign this prize-money to the Advisory Board, to be split into several smaller sums which can be given to qualified workers who can show that they have something definite and tangible in view. This is the method pursued by the British Medical Association in awarding its grants in aid of research, and it is a method which the Department will do well to consider. While the Report is necessarily vague with regard to definite schemes for dealing with patients who are already consumptive, it does well, in our opinion, to lay stress on the necessity for compulsory isolation in the case of those who are a standing danger to the community.

It will be remembered that the Final Report, with its recommendations for the prevention of tuberculosis in general, is complementary to the Interim Report of April 1912, which recommended the dispensary and the sanatorium as the two essential units in any scheme for the general treatment of the disease. The Appendix to the Report, which is published as a separate volume, contains a Memorandum on Sanatoria prepared by a Sub-Committee. It lays down the maxim that as little as possible should be spent on sanatorium construction and estimates the cost at £150 per bed.

